# Softenin, a Novel Protein That Softens the Connective Tissue of Sea Cucumbers through Inhibiting Interaction between Collagen Fibrils

**DOI:** 10.1371/journal.pone.0085644

**Published:** 2014-01-15

**Authors:** Yasuhiro Takehana, Akira Yamada, Masaki Tamori, Tatsuo Motokawa

**Affiliations:** 1 Department of Biological Sciences, Graduate School of Bioscience and Biotechnology, Tokyo Institute of Technology, Meguro-ku, Tokyo, Japan; 2 Advanced ICT Research Institute, National Institute of Information and Communications Technology, Kobe, Hyogo, Japan; University of Reading, United Kingdom

## Abstract

The dermis in the holothurian body wall is a typical catch connective tissue or mutable collagenous tissue that shows rapid changes in stiffness. Some chemical factors that change the stiffness of the tissue were found in previous studies, but the molecular mechanisms of the changes are not yet fully understood. Detection of factors that change the stiffness by working directly on the extracellular matrix was vital to clarify the mechanisms of the change. We isolated from the body wall of the sea cucumber *Stichopus chloronotus* a novel protein, softenin, that softened the body-wall dermis. The apparent molecular mass was 20 kDa. The N-terminal sequence of 17 amino acids had low homology to that of known proteins. We performed sequential chemical and physical dissections of the dermis and tested the effects of softenin on each dissection stage by dynamic mechanical tests. Softenin softened Triton-treated dermis whose cells had been disrupted by detergent. The Triton-treated dermis was subjected to repetitive freeze-and-thawing to make Triton-Freeze-Thaw (TFT) dermis that was softer than the Triton-treated dermis, implying that some force-bearing structure had been disrupted by this treatment. TFT dermis was stiffened by tensilin, a stiffening protein of sea cucumbers. Softenin softened the tensilin-stiffened TFT dermis while it had no effect on the TFT dermis without tensilin treatment. We isolated collagen from the dermis. When tensilin was applied to the suspending solution of collagen fibrils, they made a large compact aggregate that was dissolved by the application of softenin or by repetitive freeze-and-thawing. These results strongly suggested that softenin decreased dermal stiffness through inhibiting cross-bridge formation between collagen fibrils; the formation was augmented by tensilin and the bridges were broken by the freeze-thaw treatment. Softenin is thus the first softener of catch connective tissue shown to work on the cross-bridges between extracellular materials.

## Introduction

Echinoderms have unique collagenous structures called catch connective tissues or mutable collagenous tissues. The tissues can extensively change their mechanical properties, such as elasticity and viscosity, within a few minutes under the regulation of their nervous systems [1]–[3]. The tissues contain a large amount of extracellular matrix consisting mainly of collagen fibrils, proteoglycans and microfibrils [4]–[8]. The unique properties of these collagenous tissues might be due to lack of permanent associations between the collagen fibrils and the surrounding extracellular matrix because it is easy to isolate collagen fibrils from catch connective tissues, though not from the collagenous tissues of adult vertebrates [4], [5], [8]–[10]. It seems that crosslinks between the collagen fibrils and other components of the extracellular matrix are formed or broken during changes in mechanical properties. The molecular mechanisms underlying the changes are, however, not yet fully understood.

The holothurian body wall dermis is a typical catch connective tissue that shows rapid and reversible changes in its mechanical properties in response to various stimuli. Extensive studies on the dynamic mechanical properties of the dermis of the sea cucumber *Actinopyga mauritiana* revealed that the tissue can adopt three different states [11]. These are stiff, standard and soft states, which can be distinguished by elastic and viscous properties and by strain-dependent behaviors. Dermis kept in artificial sea water with normal ionic composition including Ca^2+^ (ASW) is in the standard state, that stimulated with acetylcholine or artificial sea water with a high concentration of K^+^ (KASW) is in the stiff state, and that in the Ca^2+^-free ASW (CFASW) is in the soft state.

Stiffeners and softeners of sea-cucumber dermis have been isolated from holothurian body walls. Three stiffeners have been found. One is pentapeptide NGIWYamide [12]. NGIWYamide-like immunoreactivities were found in radial nerves and nerves in body-wall dermis, which strongly suggests that this peptide is a neuropeptide that works on nerves and/or other cellular elements involved in the stiffening response [13]. The other two stiffeners are much bigger proteins, tensilin and new stiffening factor (NSF). Tensilin (*C*-tensilin), first isolated from body-wall dermis of the dendrochirotid sea cucumber *Cucumaria frondosa* [14], is a 33 kDa protein. It increases stiffness of dermis relative to the initial soft state [15]. We found a similar protein (*H*-tensilin) from the dermis of the aspidochirotid sea cucumber *Holothuria leucospilota* [10]. Although tensilins are stiffeners of soft-state dermis, they are not stiffeners of the standard-state dermis [10]. A stiffener of the standard-state dermis, NSF, which is a protein with ca. 2.4 kDa molecular mass, has been found and partially purified by us from the body-wall dermis of *H. leucospilota* [16].

Studies on softeners have lagged behind those of stiffeners. The presence of some softening factors has been shown in the coelomic fluid [17], [18] and in body wall extract [14] of sea cucumbers, but none of those has been identified. The only identified molecule with softening activity is heptapeptide holokinin that has been isolated from the body wall of *Apostichopus japonicus* [12]; however, neither its molecular mechanism nor the cells containing it are known.

The current hypothesis for the stiffness-change mechanism of catch connective tissue is as follows: some chemicals, which affect the interacting forces between macromolecules in the extracellular materials, are secreted from certain cells whose secretory activities are controlled through nerves [19]. Based on this hypothesis, the above mentioned chemical factors can be classified into two categories, those affecting nerves and/or cells involved in stiffness control and those working directly on extracellular materials in the dermis to change their stiffness. Neuropeptides NGIWYamide is in the former category. Although the studies with chemicals in the latter category are vital to the understanding of the molecular mechanism of stiffness changes, we have little information about which chemicals mentioned above belong in the latter category. This is because the effects of chemicals have been tested only on freshly isolated dermis containing live cells such as nerves and presumed stiffness-controlling cells and thus we have difficulty in determining whether a particular chemical affected the extracellular materials without affecting the stiffness-controlling cells. Tensilin may work directly on the extracellular materials because they cause aggregation of collagen fibrils when added to collagen suspending solution [10], [15]. There still remains, however, the possibility that tensilin works through affecting stiffness-controlling cells and their aggregation-inducing action has nothing to do with the dermal stiffening.

The aim of the present study was to find a softener that works directly on the extracellular materials of the sea-cucumber dermis. For this purpose we used “Triton models” of dermis in which cells have been disrupted while the extracellular components remain almost intact. We developed two kinds of Triton models, one corresponding to the standard-state dermis and the other to the soft-state dermis. Extracts from sea-cucumber body wall were fractionated through a series of columns to isolate proteins that softened the standard-state model. For the extraction, the dermis of the aspidochirotid sea cucumber *Stichopus chloronotus* was used, because its softening response is so intense that it “melts” into a slimy mass [20]. We expected that we could extract softening factors from the melted body wall that were highly active and/or present in large amounts.

We succeeded in purifying a novel protein. It softened Triton models in dynamic mechanical tests, and so it is the first softener shown to work on extracellular materials directly.

## Materials and Methods

### Ethics statement

Sea cucumbers used in present study (*Stichopus chloronotus*, *Holothuria leucospilota*) are invertebrates, thus no specific permits were required for the present study. The field studies were performed in a location that was neither privately owned nor protected. The field studies did not involve endangered or protected species.

### Animals and tissues

Specimens of the sea cucumber *Stichopus chloronotus* Brandt and *Holothuria leucospilota* Brandt were collected in Kin bay, Okinawa, Japan. They were shipped to Tokyo Institute of Technology and kept in an aquarium with recirculating sea water at 20–23°C. The dermis from the dorsal interambulacral region of holothurian body walls was isolated and used for purification of a softener, for isolation of collagen fibrils, and for mechanical tests.

Tissue samples used for the mechanical tests were Triton-treated dermis whose cells had been disrupted by Triton X-100. Triton-treatment was performed as described previously [21] with some modifications. Dermal pieces of about 2 mm×3 mm×10 mm were immersed in artificial sea water with normal composition (ASW) containing 1% (w/v) Triton X-100 (Sigma-Aldrich, St. Louis, MO, USA) at 4°C overnight for those of *H. leucospilota* and for 2 hours for those of *S. chloronotus*. The shorter treatment-time was used in *S. chloronotus* because the dermal extracellular matrix of this sea cucumber is looser than that of *H. leucospilota*. The ASW had the following composition: 0.50 mol l^−1^ NaCl, 50 mmol l^−1^ MgCl_2_, 10 mmol l^−1^ KCl, 10 mmol l^−1^ CaCl_2_ and 20 mM Tris-HCl, pH 8.0. Triton-treated dermal pieces were washed for two hours and stored at −20°C. When the stored pieces were used for mechanical tests, they were trimmed with a razor to make column-shaped samples of the desired size and then thawed in ASW. In some samples of *H. leucospilota* the Triton extraction was followed by freeze-thaw treatment in which dermis was frozen to −20°C, then thawed at room temperature in ASW; freeze-thawing was repeated at least ten times to prepare “Triton-freeze-thaw” dermis (TFT dermis). We checked whether the Triton treatment had disrupted the cellular functions by using artificial sea water with a high concentration of potassium (KASW) that had the following composition: 0.4 mol l^−1^ NaCl, 110 mmol l^−1^ KCl, 50 mmol l^−1^ MgCl_2_, 10 mmol l^−1^ CaCl_2_ and 20 mM Tris-HCl, pH 8.0. The Triton-treated dermis did not change stiffness in response to KASW. Because KASW is a potent stiffening agent that affects mechanical properties through effects on cellular activities [21], we judged that the cellular functions had been disrupted in the Triton-treated dermis. As will be shown in the discussion section, it is very likely that the Triton-treated dermis corresponded to the dermis in the standard state and the TFT dermis corresponded to the dermis in the soft state. We searched for chemicals that softened Triton-treated dermis, which thus implied that these chemicals softened standard state dermis. In addition, “fresh” dermal pieces of *H. leucospilota* which were isolated from live individuals without Triton treatment were also used for mechanical tests. We did not use fresh or TFT dermis of *S. chloronotus* because they showed such intensive strain softening that they “melted” soon after the dynamic mechanical tests had begun.

### Dynamic mechanical test

Dynamic mechanical tests were performed as described previously [10] with some modifications. A dermal column whose size was 0.5 mm×1 mm×5 mm was used. One end of the column was glued to the bottom of the trough with cyanoacrylate glue, and the other end was glued to the movable holder attached to the load cell. The long axis of the column, along which tensile strain was applied, corresponded to the long axis of the sea cucumbers. The movable holder-load cell complex was attached to a vibrator that was driven by triangular electric pulses of 0.3 Hz. The holder moved up to stretch the column by + 5% of the initial length and then it moved down, with the same speed as in the upward movement, to push the column to −5%.

Tensile force was monitored by a load cell. The maximal stiffness of the sample in one stretch-compression cycle was proportional to the maximal tensile stress which was used as a measure of stiffness. Otherwise stated, it was expressed as a relative value in percent, normalized by the peak value in the steady state as 100% (see RESULTS).

After the dermal sample was fixed in the experimental trough, 50 µl of ASW was introduced. We started measurements 10 min after ASW introduction. A test solution that contained softener or *H*-tensilin was introduced during the steady phase that will be defined in RESULTS. ASW in the trough was replaced with the test solution whose ionic composition was adjusted to the same one as that of ASW. The final concentration of the purified softener was adjusted to 60 µg ml^−1^. The mechanical tests were performed at room temperature (20–24°C), which did not change by more than 1°C during any one mechanical test.

### Screening of fractions for softening activity

We tested each fraction eluted from chromatography columns by dynamic mechanical test on the Triton-treated dermis of *S. chloronotus* to find out fractions containing the softener. When relative stiffness decreased to less than 80% of that just before the application of a fraction in 10 min, we regarded that the fraction contained the softener.

### Purification of softener

Twenty gram dermal pieces were placed in the same volume of 5 mol l^−1^ NaCl solution containing 20 mmol l^−1^ Tris-HCl (pH 8.0) at 4°C and stirred with a stainless steel bar for two hours. This procedure caused the dermis to “melt” into a homogenate, which very probably corresponded to the melting of body walls when this sea cucumber species was subjected to strong mechanical stimuli [20]. The homogenate was centrifuged at 28,000×g for 40 min at 4°C. The supernatant was dialyzed against 10 mmol l^−1^ Tris-HCl, pH 8.0 and filtered through the micro-filtration device Vivaspin 2 (Molecular weight cut off = 1,000,000; Sartorius AG, Göttingen, Germany) to remove viscous materials. The filtrate thus obtained was the crude extract, which was applied to a Mono-Q chromatography column (Pharmacia Biotech, Piscataway, NJ, USA) equilibrated with the same buffer as the dialysis solution. The column was washed with the same buffer containing no NaCl, and eluted with the buffer containing increasing concentration of NaCl (the stepwise elution 0 and 0.1 mol l^−1^ followed by the gradient elution 0.1–1.0 mol l^−1^). The fractions from the stepwise elution were pooled and applied on two serially-attached gel-filtration columns of Superose 12 10/300 GL (Pharmacia Biotech) equilibrated with 0.5 mol l^−1^ NaCl and 20 mmol l^−1^ Tris-HCl, pH 8.0. The fractions with softening activities were collected, and concentrated with ultra-filtration device Vivaspin 500 (Molecular weight cut off = 3,000; Sartorius AG). Concentrated sample was applied on a Superdex Peptide 10/300 GL gel-filtration column (Pharmacia Biotech) equilibrated with the same solution as used in Superose 12 column, and the purified softener fraction was collected. The concentration of the softening protein was measured with Qubit protein assay kit (Life Technologies, Carlsbad, CA, USA) using bovine serum albumin as a standard.

Sodium dodecyl sulfate polyacrylamide gel electrophoresis (SDS-PAGE) was performed according to Schägger and von Jagow [22], using 11% gels under the reducing condition. The gels were stained with Coomassie Brilliant Blue R-250 to visualize proteins. To analyze the N-terminal amino acid sequence, proteins in the gel were transferred to PVDF membrane. They were degraded by Edman degradation and fragments were determined with a protein sequencer Procise cLC (Applied Biosystems, Foster City, CA, USA). The obtained sequence was compared with other known protein sequences using BLAST (www.ncbi.nlm.nih.gov).

### Purification of *H*-tensilin

*H*-tensilin was purified from the dermis of *H. leucospilota* as described previously [10] with some modifications. In brief, the dermis was homogenized in 2 mol l^−1^ NaCl, 10 mmol l^−1^ EGTA and 20 mmol l^−1^ Tris-HCl, pH 8.0. Its supernatant after centrifugation was precipitated with 60% saturated (NH_4_)_2_SO_4_. The precipitate was dissolved in 0.5 mol l^−1^ NaCl and 20 mmol l^−1^ Tris-HCl, pH 8.0, and dialyzed against the same solution. The crude extract was then applied to a Mono-Q chromatography column equilibrated with the same solution. Stepwise elution fractionation between 0.65 mol l^−1^ and 1.0 mol l^−1^ NaCl was used for purification of *H*-tensilin. This fraction was further applied on a Superose 6HR 10/30 gel-filtration column (Pharmacia Biotech) equilibrated with 0.5 mol l^−1^ NaCl and 20 mmol l^−1^ Tris-HCl, pH 8.0, and the *H*-tensilin fraction was collected. *H*-tensilin concentration was measured with Qubit protein assay kit.

### Aggregation assay

Collagen fibrils were isolated from the dermis of *H. leucospilota*, and the aggregation assay was performed in buffer without Ca^2+^ (0.5 mol l^−1^ NaCl, and 20 mmol l^−1^ Tris-HCl, pH 8.0) as described previously [10] with some modifications. Tensilin induced collagen aggregation (see Results). We studied the effect of the softener on the tensilin-aggregated collagen fibrils. In test samples, the softener was applied to a suspending solution of collagen fibrils that had been previously aggregated by application of *H*-tensilin. In control samples, buffer without Ca^2+^ was added to the suspending solution instead of the softener. The final concentrations of the softener and *H*-tensilin were 11.8 µg ml^−1^ and 20 µg ml^−1^ respectively which were adjusted to the same number of molecules of these two agents in the suspending solution. We also studied the effect of repetitive freeze-thawing on the tensilin-induced aggregation. In test samples, the solution with the aggregate was frozen to −20°C, and then thawed under room temperature; the freeze-thawing was repeated three times. In control samples, solution with the aggregate was stored at 4°C while test samples were under freeze-thaw treatment which took about 24 hours.

## Results

### Purification of softener

**Figure 1:**
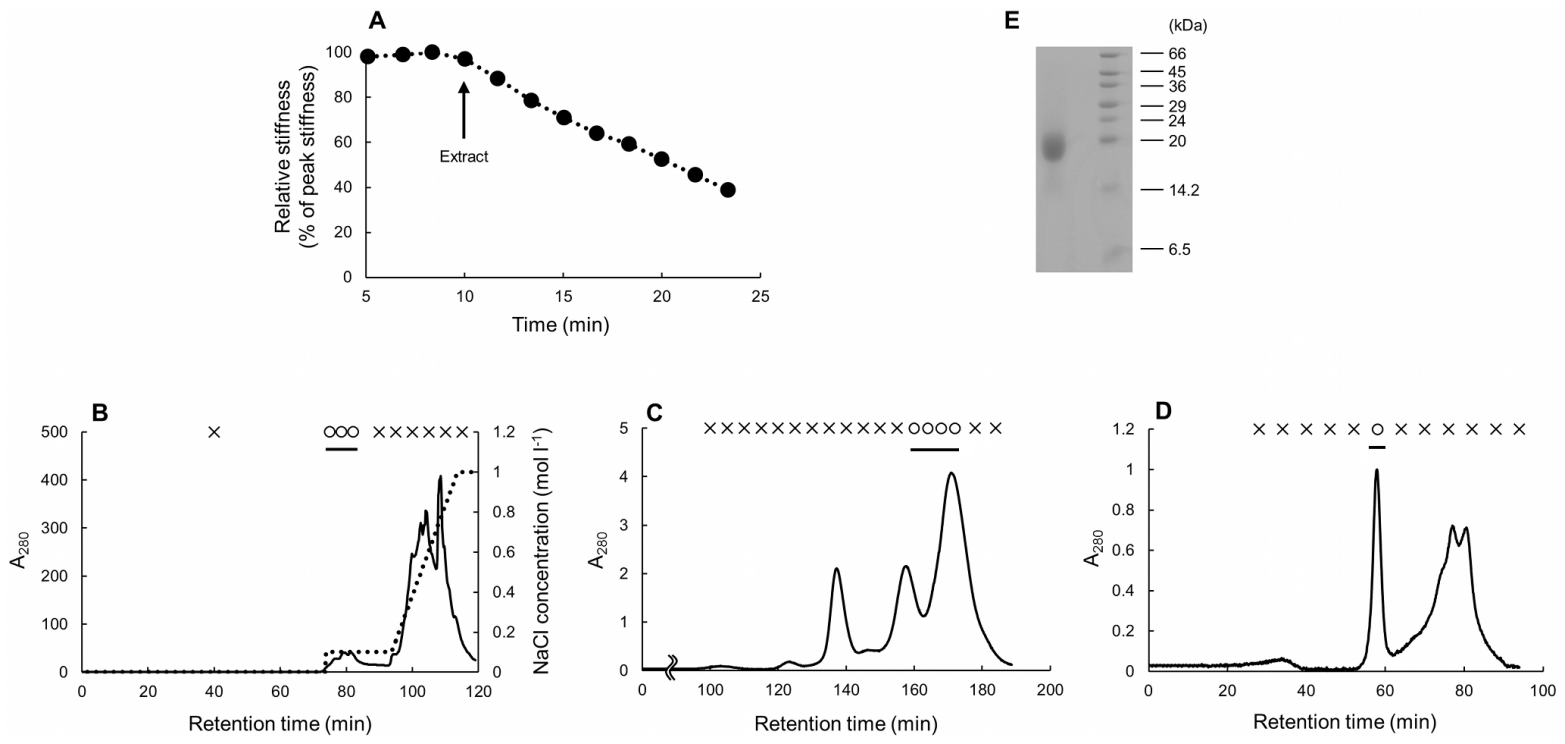
Screening and purifying of the softener. (A) The result of the mechanical tests on the Triton-treated dermis of *S. chloronotus*. In this and Figs 3 and 4 the ordinate is relative stiffness normalized by the peak value in the steady phase of the respective experiment. The application of the crude extract (upward arrow) caused a decrease in stiffness. (B) Anion-exchange chromatography on a Mono-Q column of an extract of sea cucumber dermis. The solid line shows the absorbance at 280 nm; the dotted line shows NaCl concentration. In B-D the fraction marked with a circle had softening activity and that with a cross showed no softening activity, and a horizontal bar indicates the fractions processed for further screening. The left-hand-side ordinate of graphs B-D is given in relative values in which the absorbance of the purified softener, the peak value in panel D, was taken as unity. (C) Gel-filtration chromatography on the two serially-attached Superose 12 columns of the active fractions from the Mono-Q column. (D) Gel-filtration chromatography on the Superdex-peptide column of the active fractions from the Superose 12 columns. (E) The result of SDS-PAGE of the active fraction from Superdex peptide column (left lane). The right lane is molecular weight markers.

The crude extract had softening activities (Fig. 1A). The relative stiffness 10 min after the application of the extract was 54.0% of that just before the application of the extract (one trial). The results of the purification process through a series of column chromatographies are shown in Fig 1. By Mono Q column, the softener was found in the fractions eluted between 0–0.1 mol l^−1^ NaCl (Fig. 1B). By Superose 12 column, it was found in the fractions that composed a peak with the longest retention time and the highest A_280_ (Fig. 1C). By Superdex-peptide column, softening activities were found in the first large and sharp peak of A_280_ (Fig. 1D); SDS-PAGE of the active fraction gave a single band (Fig. 1E) whose apparent molecular mass was approximately 20 kDa. Thus we regarded this molecule as the softener, and the active fraction obtained through three-step column chromatography was used as the softener in the following experiments. The yield of the softener was about 85 µg from 20 g dermis. Although we performed mechanical tests of all the fractions eluted from columns, we found no fraction with stiffening activities on Triton-treated dermis of *S. chloronotus*.

**Figure 2:**
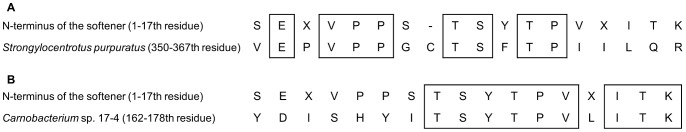
Comparison of amino acid sequences between the softener and other known proteins by BLAST. In N-terminal amino acid sequence of the softener, third X residue means unidentified residue and 14th X residue means proline or phenylalanine. The third residue was excluded in calculation of the identity. Identical residues were boxed. (A) Sequence comparison between the softener and partial amino acid sequence of ubiquitin carboxyl-terminal hydrolase 34-like protein from *Strongylocentrotus purpuratus* (XP_785288.3) that showed the highest bit score among echinoderms. Identity was 47.1%. (B) Sequence comparison to partial amino acid sequence of GntR family transcriptional regulator from *Carnobacterium* sp. 17-4 (YP_004375176.1) that showed the highest bit score among whole organism. Identity was 56.3%.

The N-terminal sequence of 17 amino acids of the softener was SEXVPPSTSYTPVXITK. The third residue could not be detected because it had been modified; the 14th residue was either proline or phenylalanine. This sequence was compared to those of other known proteins by BLAST. Among echinoderms the proteins with the top 5 highest similarity bit scores were, from the higher to lower, ubiquitin carboxyl-terminal hydrolase like protein (24.4 bits), minichromosome maintenance 3-associated protein, HES family transcription factor of starfish, tumor necrosis factor receptor, and 4-hydroxyphenylpyruvate dioxygenase. All proteins but HES family transcription factor were derived from sea urchin *Strongylocentrotus purpuratus* whose genome has been read already. Among whole organism the proteins with the top 5 highest bit scores were, from higher to lower, GntR family transcriptional regulator of *Carnobacterium* (31.2 bits), nuclear hormone receptors-153, mitochondrial 2-oxoglutarate/malate carrier protein and two amyloid-like proteins, all of which had higher bit scores than those of echinoderms. Proteins with the highest bit scores among whole organism and among echinoderms were compared to the softener by alignment those sequences (Fig. 2). The identity of the bacterial protein was 56.3% and the sea-urchin protein was 47.1%.

### Dynamic mechanical tests

#### Triton-treated dermis of *Stichopus chloronotus* (S-dermis)

**Figure 3:**
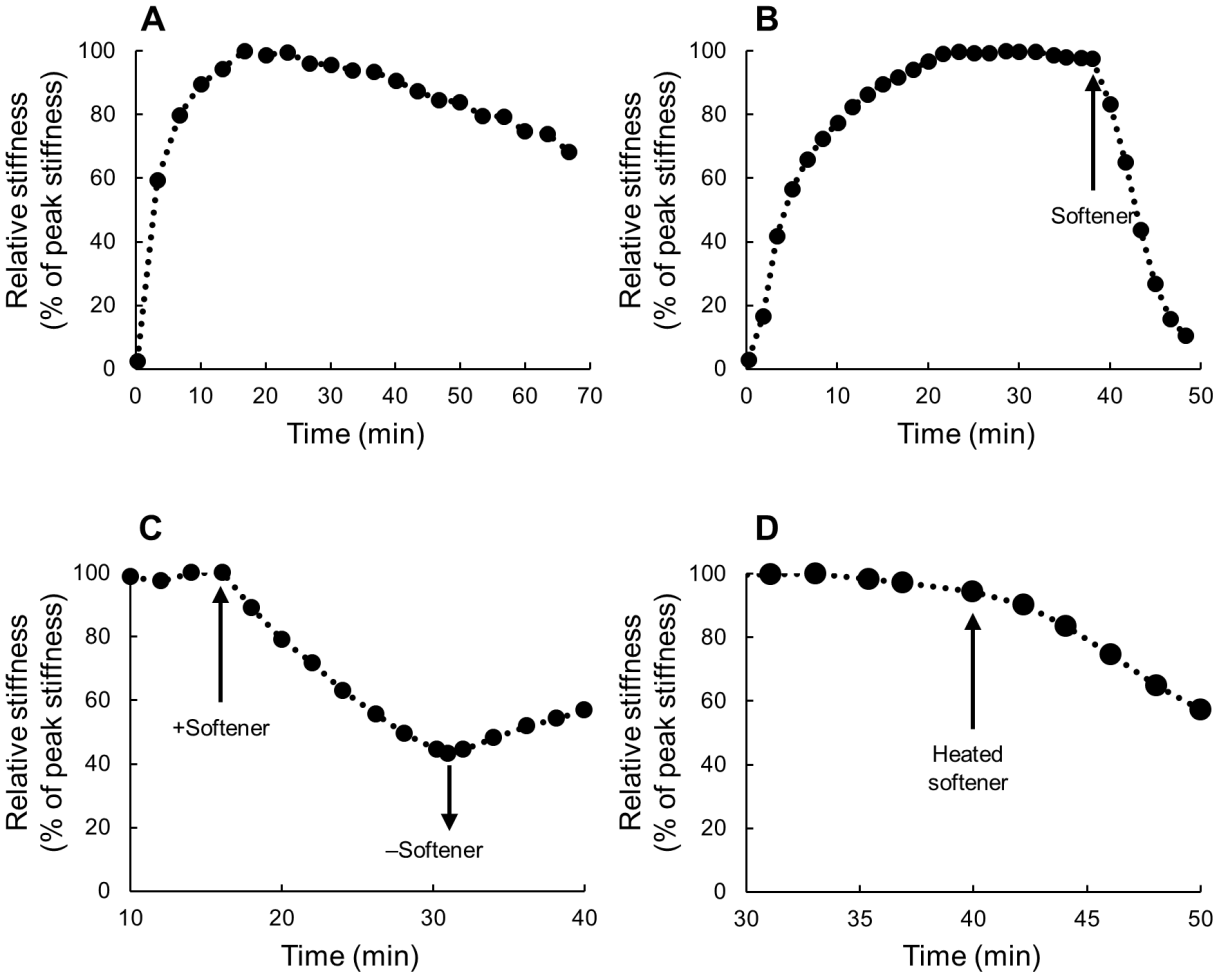
Typical results of the mechanical tests on the Triton-treated dermis of *S. chloronotus*. (A) A control in which the stiffness measurement was performed in ASW for more than 1 hour without application of the softener to show the initial phase of rapidly increasing stiffness and the following steady state phase. (B) The effect of the softener. The application of the softener (upward arrow) caused a rapid decrease in stiffness. (C) Recovery experiments after the application of the softener. Stiffness slightly recovered by washing out the softener with ASW (downward arrow). (D) The effect of the heated softener. The application of the heated softener (upward arrow) caused a decrease in stiffness.

Stiffness rapidly increased at the start of all dynamic mechanical tests. The increase was apparent in 5–15 seconds that corresponds to the second to the fourth stretch-compression cycles. In 10–50 min (180th-900th cycles) stiffness reached a peak that was several times larger than the stiffness value just after the test had begun (Table 1). Subsequently, the peak value decreased slowly and slightly (Fig. 3A). Thus the dermis showed two phases, the early rapidly increasing phase and the late almost steady phase. No spontaneous rapid changes in stiffness were observed during the steady phase. Softener at the concentration of 60 µg ml^−1^ caused rapid decrease in stiffness. Figure 3B shows a typical example in which the decrease became apparent about 20 seconds after the application of the softener, and in 10 min stiffness decreased to the value less than one-fifth of that just before the application of the softener in most samples. Stiffness remained decreased as long as the softener was present. The relative stiffness 10 min after the application of the softener was 17.9±10.5% of that just before the application of the softener (mean±s.d., n = 7) whereas that of control, which was 10 min after the introduction of ASW without the softener, was 93.2±3.9% of that just before the introduction of ASW (n = 5). These two medians were statistically different (P<0.01) by U-test. The softener was also effective at a lower concentration of 20 µg ml^−1^. The relative stiffness 10 min after the application of the softener was 34.7% of that just before the application of the softener (one trial). Stiffness recovered partially when the softener was washed out with ASW (Fig. 3C). The recovery was apparent within a minute after washing. The softener that had been heated at 80°C for 20 min, still softened Triton-treated dermis (Fig. 3D); the relative stiffness 10 min after the application of the heated softener was 60.5% of that just before the application of the softener (one trial).

**Table 1:** The stiffness (kPa) of dermal preparations†.

			*H. leucospilota*			*S. chloronotus*
Time§	TFT		Triton-treated	Fresh		Triton-treated
0 min	9.05±1.67 (5)		8.68±5.02 (9)	8.60±5.57 (7)		8.3±3.5 (10)
15 min	8.16±1.55 (5)		69.89±51.99* (9)	48.14±7.38* (7)		79.0±41.39* (10)

Values are mean ± s.d. (number of experiments).

Time in minutes after the beginning of dynamic tests.

Statistically different from TFT dermis (P<0.05).

### *Effects on dermis of* Holothuria leucospilota (H-*dermis*)

**Figure 4:**
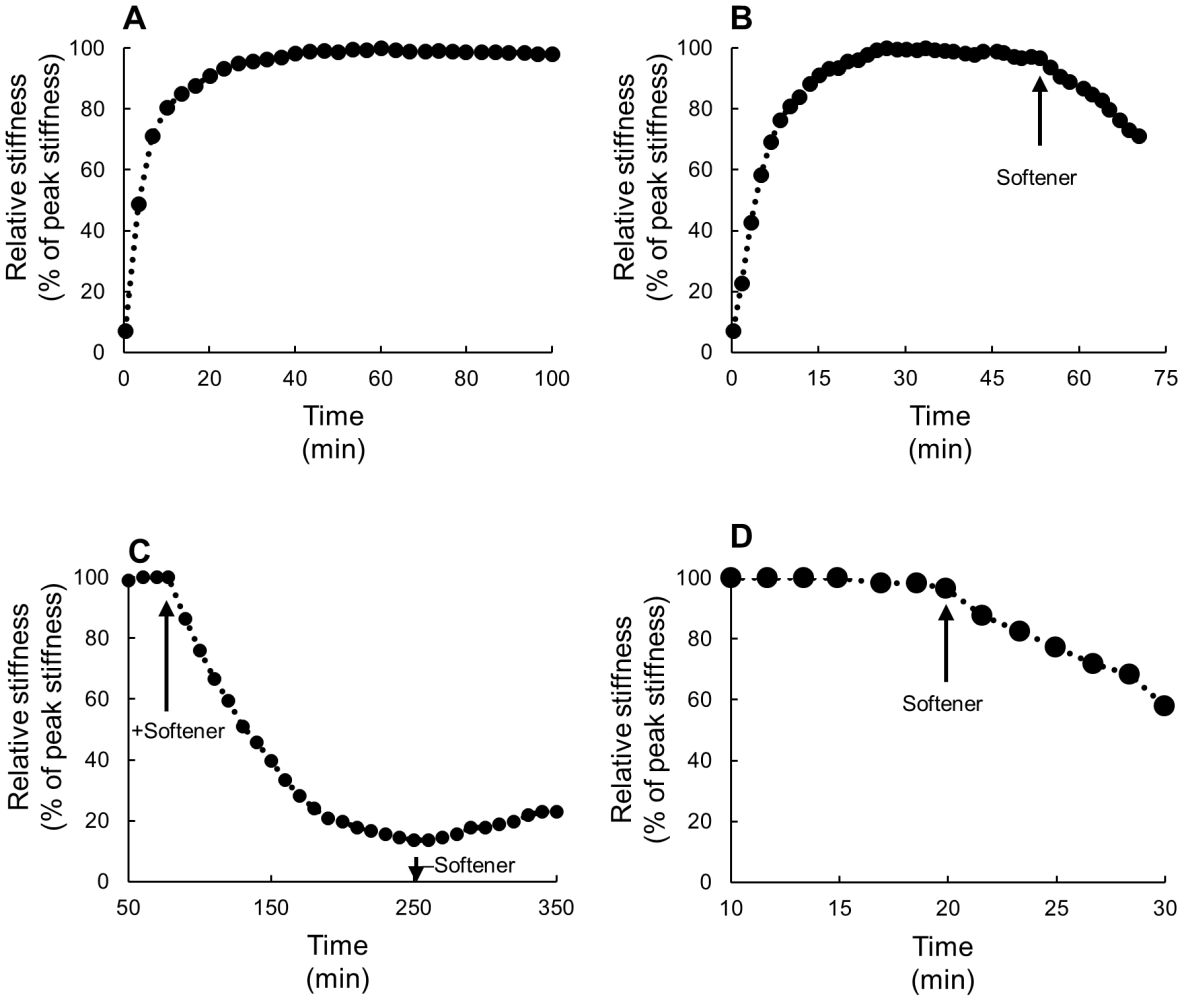
Typical results of the mechanical tests on the dermis of *H. leucospilota*. (A–C) The Triton-treated dermis of *H. leucospilota*. (A) A control. Two phases could be seen as in *S. chloronotus*. (B) The effect of the softener showing that it softened the dermis of this species also. (C) Recovery experiments after the application of the softener. Stiffness slightly recovered by washing out the softener with ASW (downward arrow). (D) Fresh dermis of *H. leucospilota*. The application of the softener (upward arrow) caused a decrease in stiffness.

Softener was effective even on dermis taken from a different species, *H. leucospilota*. The Triton-treated *H*-dermis also showed two phases, the early rapidly increasing phase and the later steady phase, in dynamic tests; the peak value of the latter phase was held for more than an hour (Fig. 4A). The introduction of the softener in the steady phase caused a decrease in stiffness (Fig. 4B). The relative stiffness 10 min after the application of the softener was 74.42±10.9% of that just before the application of the softener (mean±s.d., n = 4). The stiffness of Triton-treated *H*-dermis showed partial recovery when the softener was washed out with ASW (Fig. 4C) as had *S*-dermis. The recovery was, however, slower; it was apparent 10 min after washing. The softener caused stiffness decrease of fresh *H*-dermis (Fig. 4D) when applied after having attained the steady phase that followed the early rapidly increasing phase. The relative stiffness 10 min after the application of the softener was 68.0±8.7% of that just before the application of the softener (mean±s.d., n = 3). There was no significant difference between the mean relative stiffness of Triton-treated dermis and that of fresh dermis by t-test (P>0.05).

**Figure 5:**
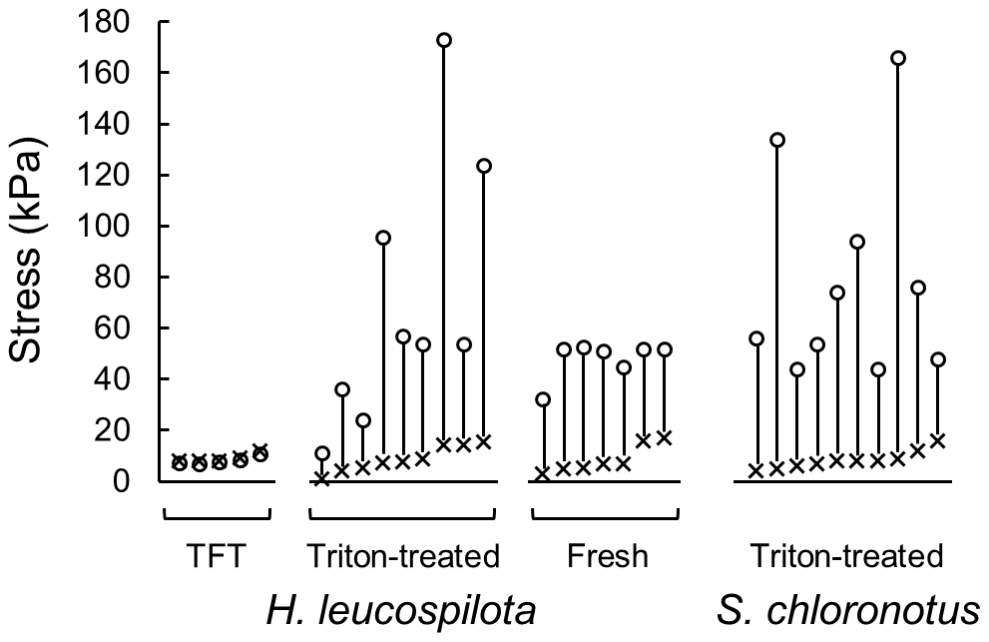
Responses to vibration imposed in dynamic tests. Three different kinds of dermal preparations, TFT, Triton-treated for two species, and fresh, were compared. The right-most column is for *S. chloronotus* and the other three are for *H. leucospilota*. The values of stress at +5% strain just at the beginning of a dynamic test (cross) and 15 minutes after the test had started (circle) in a single dynamic test were connected with a vertical bar. Notice each circle is a little below the corresponding cross in TFT whereas it is high above in Triton-treated dermis and in fresh dermis.

**Figure 6:**
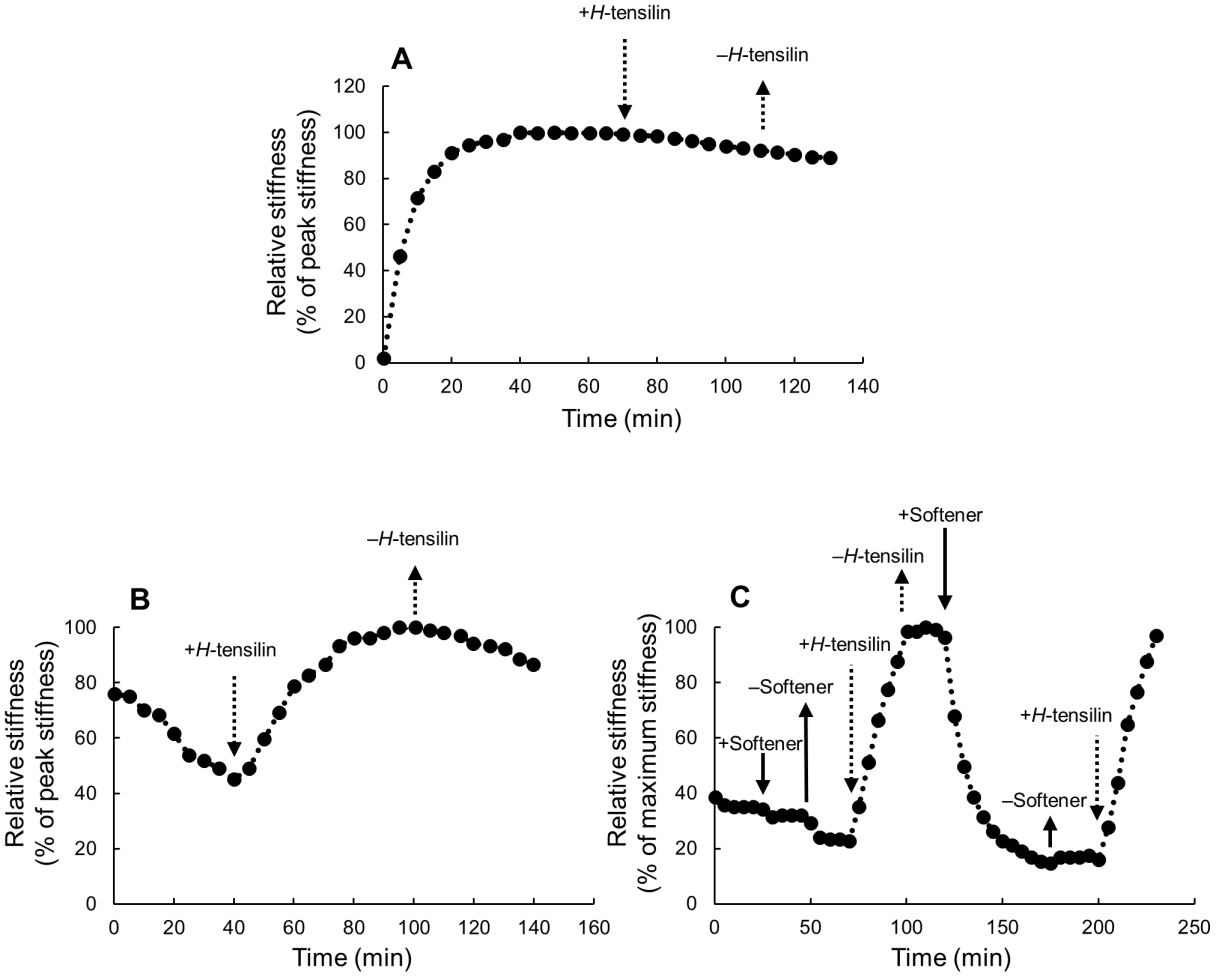
Effects of *H*-tensilin and the softener on the Triton- freeze-thaw (TFT) dermis of *H. leucospilota*. The ordinate is the relative stiffness that was normalized by the peak value found in tensilin as 100%. The arrows show introduction (downward ones) or removal (upward ones) of agents. The agents were removed by draining off the solution with agents from the trough followed by the introduction of ASW without agents. The solid arrows denote the softener and the broken arrows denote tensilin. (A) *H*-tensilin (3 µg ml^−1^) did not cause stiffening of the Triton-treated dermis of *H. leucospilota*. (B) The effect of *H*-tensilin (3 µg ml^−1^) on stiffness of the TFT dermis of *H. leucospilota*. In the TFT dermis stiffness continued decreasing slowly and slightly during the whole testing period. The introduction of tensilin induced a prominent stiffness increase. (C) Results of the combination experiments of *H*-tensilin (10 µg ml^−1^) and the softener (60 µg ml^−1^). The softener did not cause softening of the TFT dermis but it softened the dermis stiffened by tensilin beforehand.

The TFT dermis behaved differently from Triton-treated dermis and fresh dermis both in response to the vibration imposed by the testing machine (Fig. 5) and in the response to the softener (Fig. 6C). The TFT dermis did not stiffen at the start of the dynamic testing. Stiffness, however, continued decreasing slowly and slightly from the start to the end of the testing and thus stiffness remained low when no chemical stimulation was applied (Figs. 5, 6B, C, Table 1). When softener was applied on such soft dermis no further softening was observed (Fig. 6C).

Figure 5 and Table 1 summarize stiffness of various kinds of dermal preparations we used including that of *S. chloronotus*. Stiffness was represented by the peak stress at + 5% strain in a push-pull cycle. Stiffness values at the beginning of tests were similar in all preparations: they were about 9 kPa. The stiffness values after 15 min of vibration were, however, quite different. The stiffness decreased a little in every TFT dermis to 8 kPa on average while in the other three kinds of dermis it greatly increased to 50–80 kPa. Steel-Dwass multiple comparison of stiffness values at 15 min of 4 kinds of dermis showed that there was a significant difference between TFT dermis and the other three but no difference among the other three at the level P = 0.05.

#### Combination experiments of tensilin and softener on TFT dermis

*H*-tensilin is a protein isolated from the body wall of *H. leucospilota*. Although tensilin at the concentration 3 µg ml^−1^ does not stiffen fresh dermis in ASW, it stiffens dermis already softened in CFASW [10]. We found that tensilin at the same concentration in ASW did not stiffen Triton-treated *H*-dermis (Fig. 6A). In the TFT dermis, however, tensilin at the same concentration (Fig. 6B) or higher (3–10 µg ml^−1^) caused marked stiffening. The increased stiffness was maintained or stiffness decreased only slowly and slightly when tensilin was washed out by ASW. Figure 6C is the result of the combination experiment in which the softener (60 µg ml^−1^) and tensilin (10 µg ml^−1^) were applied successively. We first showed that the softener had no effect on the TFT dermis. Then the dermis was stiffened by tensilin. The introduction of the softener to this stiffened dermis caused a rapid decrease in stiffness to the level similar to that before tensilin application. The second application of tensilin after having washed out the softener increased stiffness again to the level comparable to that attained by the first application of tensilin (Fig. 6C).

### Collagen fibril aggregation assay

**Figure 7:**
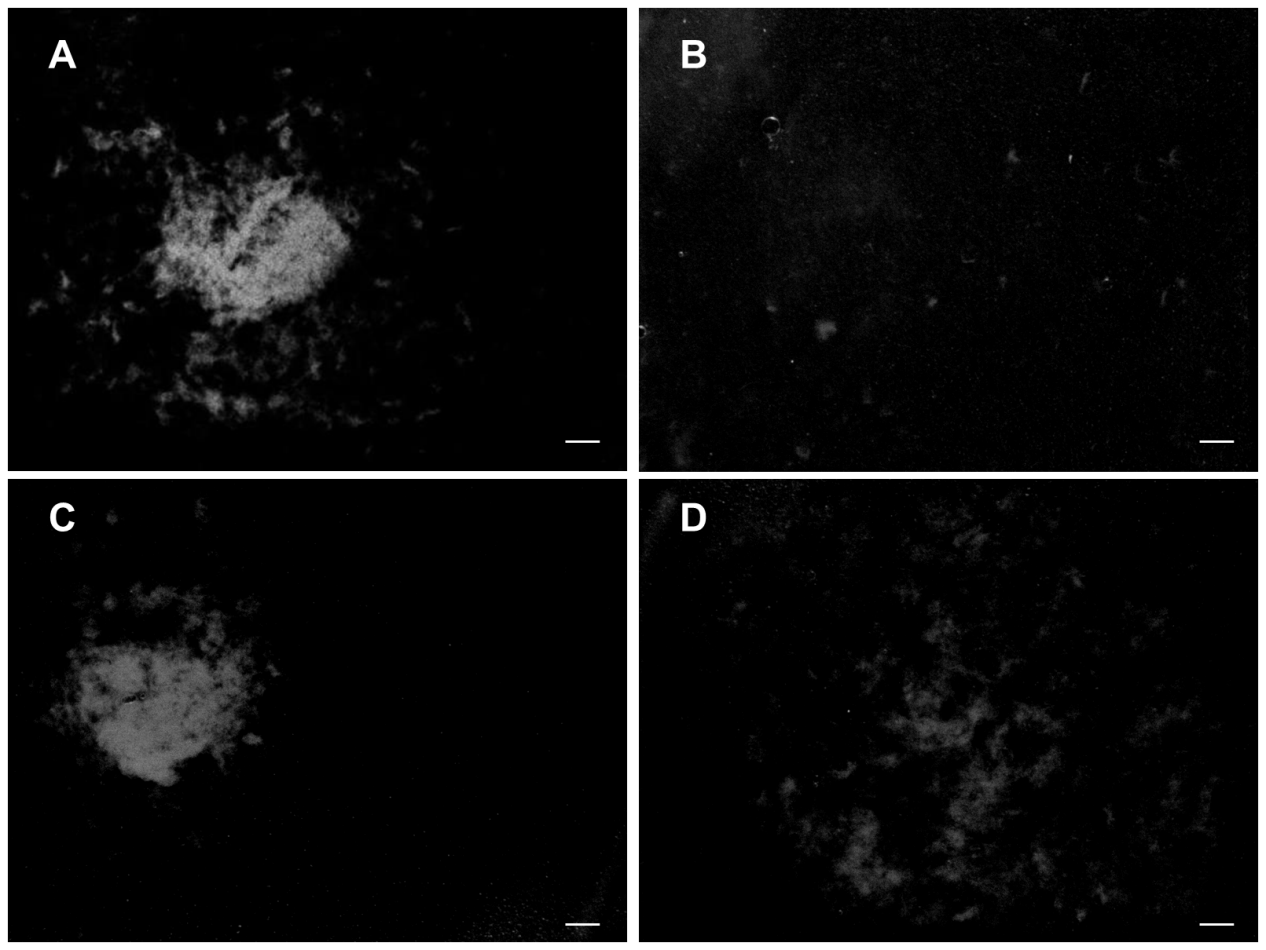
Results of the aggregation assay using collagen fibrils from the dermis of *H. leucospilota*. (A,B) The effect of the softener and (C,D) the effect of the repetitive freeze-thaw treatment on aggregation induced by tensilin. Samples were observed under a light microscope. Scale bars, 1 mm. (A) *H*-tensilin caused a large compact aggregate when applied to the suspending solution of collagen fibrils. (B) The introduction of the softener to the solution with a tensilin-induced large aggregate. The aggregate dissolved into small fluffy aggregates. (C) Control to D. The tensilin-induced aggregate was maintained in the solution with tensilin when it was stored at 4°C. (D) After the repetitive freeze-thaw treatment, the large compact aggregate dissolved into small fluffy aggregates.

*H*-tensilin caused aggregation of collagen fibrils as was reported previously [10]. It caused a single large compact clot under a microscope (Fig. 7A), whereas the softener caused no aggregates. When the softener was added to the aggregated-collagen fibrils, the large compact aggregate was dissolved into many small fluffy aggregates (Fig. 7B). The introduction of buffer solution alone did not cause such dissolution. When the tensilin-induced aggregate was subjected to the repetitive freeze-thaw treatment, the large aggregate also dissolved into small fluffy aggregates (Fig. 7D).

## Discussion

We purified a protein from the sea cucumber *Stichopus chloronotus* that softened the dermis in its body wall. Its N-terminal amino acid sequence was determined. When this sequence was compared to those of other known proteins, we found only one protein that might possibly soften the connective tissue and which had high similarity bit scores and high identity. The hydrolase of a sea urchin may soften by digestion. Our present results, however, showed that the action of the softener was not through simple digestion (as will be discussed later). Thus we regarded that the softener is novel protein. We coin the name “softenin” after the function of this new protein. Because softenin of *S. chloronotus* was also effective on *H. leucospilota*, softenin and its homologues are likely common softeners in sea cucumbers.

We used Triton-treated dermis whose cells had been disrupted by the detergent Triton X-100 for bioassay in order to find chemicals that exerted their effects on the mechanical properties of extracellular materials. As the stiffness value of Triton-treated dermis was not different from that of fresh dermis in the standard state, it is very likely that Triton-treated dermis corresponded to the dermis in the standard state; it was not in the stiff state, which is much stiffer [10]; it was also not in the soft state, which will be discussed in the next paragraph. Softenin decreased stiffness of both Triton-treated dermis and fresh dermis, which implies that softenin was effective on the dermis in the standard state irrespective of whether cells inside were alive or not. Thus softenin is the first chemical, other than inorganic ions [21], shown to directly change the stiffness of extracellular materials.

We tried to make a cell-disrupted “model” dermis that corresponded to the dermis in the soft state. We added freeze-thaw treatment to Triton-treated dermis expecting that this extra freeze-thaw treatment might disrupt some mechanical interactions between macromolecules to make the dermis softer. TFT dermis had lower stiffness as was expected. We inferred that TFT dermis corresponded to the soft-state dermis not only because it was softer but also because it showed softening by repetitive straining (Fig. 5, Table 1). Further contributing to this inference was that it showed stiffening by tensilin (Fig. 6B). The increase in stiffness at the start of dynamic tests was observed in fresh dermis as in previous studies [10], [23] and in Triton-treated dermis but not in TFT dermis. In the holothurian dermis collagen fibers make a three-dimensional array. Repetitive uniaxial straining imposed on such fibrous materials makes fibers align in the direction of straining which stiffens the materials in that direction [24]. This strain stiffening possibly accounts for a part of the initial stiffening observed both in fresh dermis and in Triton-treated dermis. Although the aligning of fibers very probably had occurred also in TFT dermis, it showed softening rather than stiffening. Strain softening was one of the features of the soft state [11]. Thus the initial softening of TFT dermis suggested that such strain softening, large enough to overwhelm the strain stiffening by aligning, occurred, which supported the view that TFT dermis was in the soft state. This view was further supported by the result with tensilin. Tensilin is a sea-cucumber derived protein that stiffens the soft dermis but does not stiffen the standard dermis [10]. The present result that tensilin was effective on TFT dermis (Fig. 6B) but not on Triton-treated dermis (Fig. 6A) suggested that TFT dermis was in the soft state and Triton-treated dermis was in the standard state. The present result also provides definitive evidence that tensilin works directly on the extracellular materials although this had already been surmised when its collagen-aggregation-inducing action was discovered [15]. Because the previous experiments used only fresh dermis in which cells were alive, it could not be determined whether agents exerted effects directly on extracellular materials or indirectly by affecting activities of cells controlling extracellular stiffness. Such discrimination is crucial for the understanding of both molecular mechanisms and neural controlling mechanisms. Thus the cell-disrupted dermal models we have developed will be of great help in the studies on catch connective tissue.

**Figure 8:**
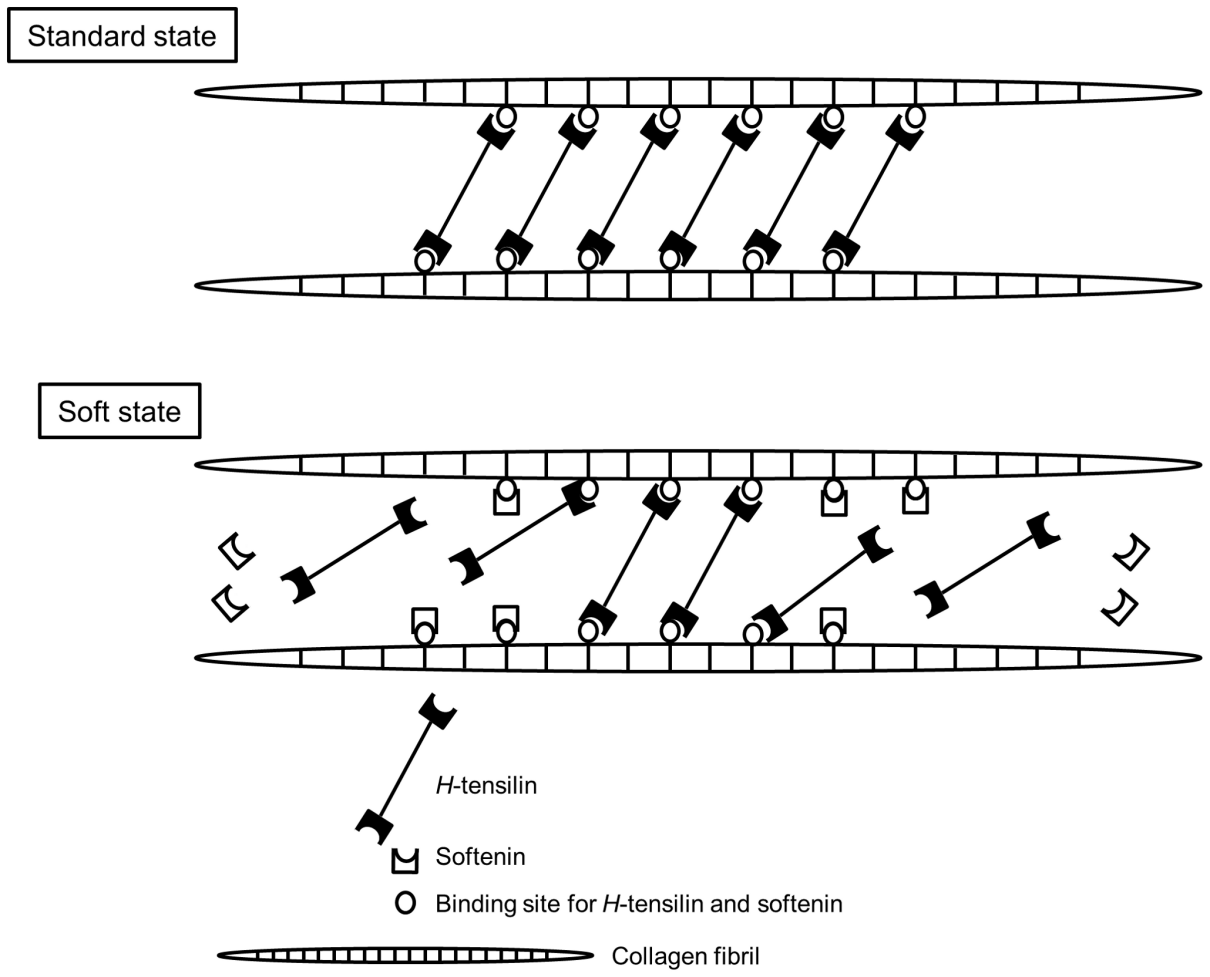
Hypothetical model of stiffness change mechanism. In this model the factors that are involved in the stiffening from the standard state to the stiff state are omitted.

Softenin had no effect on TFT dermis, which is a reasonable result if softenin was a softener of the standard dermis but not of the already soft dermis. TFT dermis stiffened by tensilin was softened by softenin only to the stiffness value before the application of tensilin, which implied that softenin cancelled the stiffening action of tensilin (Fig. 6C). The aggregation assay of collagen showed that softenin dissolved the aggregation induced by tensilin (Fig. 7B). These results strongly suggested that tensilin and softenin were working antagonistically on the same mechanical interaction between collagen fibrils. This mechanical interaction, which was associated with the change between the soft state and the standard state, was weakened by the repetitive freeze-thaw treatment. The susceptibility to the freeze-thaw treatment was also observed in the collagen suspending solution. A large compact aggregate of collagen induced by tensilin dissolved into many small fluffy aggregates by this treatment (Fig. 7D). Freezing may push two interacting collagen fibrils apart by the formation of ice between them, which imposes tensile forces on the interfibrillar bonds. Those forces may directly break the bonds or they may induce stretch softening that is characteristic to the soft state of intact dermis [11]. The ice formation may increase the concentration of ions in the non-freezing space between fibrils, which breaks the interfibrillar bonds because high ionic strength is known to make intact dermis soft [25]. The freeze-thaw treatment may oxidize cysteine residues, which may affect the interaction of fibrils; Oxidation, however, induces fibril aggregation [26], which was contrary to our results, and thus this mechanism is unlikely although there remain possibilities that the freeze-thaw treatment may induce other chemical changes that could affect collagen and tensilin. Our results suggested that both softenin and the freeze-thaw treatment could release the mechanical interaction between collagen fibrils formed by tensilin through the mechanism that is possibly shared with the softening mechanism of intact dermis as has been discussed above. Therefore here we propose a very simplified model for the stiffness change from standard to soft in which only collagen, *H*-tensilin, and softenin are involved. *H*-tensilin is hypothesized to form crossbridges between collagen fibrils and softenin is supposed to compete for the binding sites of *H*-tensilin to collagen (Fig. 8).

Some sea cucumbers have been known to show extensive softening of body walls in which they become a viscous mass in response to variety of environmental and mechanical factors [20], [27], [28]. Digestive enzymes have been proposed to cause such extensive softening [28] and a matrix metalloproteinase (MMP) that digests collagen was found in the body wall of the sea cucumber *Apostichopus japonicus* [29]. The action of softenin is, however, not likely to be simple digestion of extracellular materials such as collagen. The effect of softenin was apparent within a minute and stiffness recovered also within a minute, although partially, by removal of softenin. Such a rapid and reversible response is hard to explain by a simple enzymatic digestion because the digestion of collagen took hours [29]; re-synthesis of collagen may also take time and requires living cells such as fibroblasts which did not exist in our present system. Ribeiro et al. [30] proposed a stiffness-change mechanism in which MMPs are involved. In their hypothesis MMP is supposed to digest not collagen itself but crosslinks between collagen fibrils and the digestion is controlled by the tissue inhibitor of MMP (TIMP). This hypothesis is based on the result that tensilin has 21–36% homology to the vertebrate TIMP, although TIMP activity is yet to be shown and disulfide bonds necessary for TIMP activity are lacking in *H*-tensilin [10]. In this hypothesis MMP digests crosslinks to make the tissue soft; the recovery from soft to standard requires new supplies of both crosslinks and TIMP from cells in the tissue, which is impossible in the present experimental systems. Although our present result also suggested the antagonism between softenin and tensilin, it did not support the idea that softenin is a digestive enzyme. The possibility remains, however, that MMP may take part in the extensive softening phenomena of autotomy and fission. Complete amino acid sequencing is underway. We are now determining more amino acid sequences from the tryptic fragments of softenin. We are planning to make primers based on these fragments and to extract mRNA from body wall dermis of *S. chloronotus*, which will be used for RT-PCR to obtain the objective cDNA fragments. We will analyze its total base alignment to clone coding DNA of softenin. Another strategy we are going to adopt after having succeeded in sequencing several tryptic fragments is to find a softenin-like protein in the genome of the *S. purpuratus*. Catch connective tissue is found also in sea urchins and thus we can expect the presence of the sequence of softenin-like protein in the genome. Although we could not find the sequence with high similarity scores in the already characterized proteins, more sequence data than the present short N-terminal sequence might pick up the whole sequence of sea-urchin softenin from the characterized and uncharacterized sea-urchin proteins.
